# Overexpression of LINC00936 Inhibits the Proliferation and Invasion of Endometrial Carcinoma Cells

**DOI:** 10.1155/2022/2223954

**Published:** 2022-05-31

**Authors:** Haizhu Sun, Peiling Li

**Affiliations:** Department of Gynecology and Obstetrics, Second Affiliated Hospital of Harbin Medical University, Harbin 150001, China

## Abstract

**Objective:**

Endometrial carcinoma (EC) is one of the most common malignancies leading to death in women and poses a serious threat to women's health. Therefore, exploring the molecular mechanisms affecting EC progression and metastasis is a clinical research hotspot. It has been shown that lncRNAs play an important role in the pathogenesis of EC. It is important to investigate the role of lncRNAs in the growth of EC to improve diagnosis and find new therapeutic targets of EC.

**Methods:**

The expression of LINC00936 in 36 EC tissues, paracancerous tissues, and cell lines was measured by fluorescence quantitative PCR. The relationship between LINC00936 expression and clinicopathological characteristics of patients was analyzed. The effects of overexpression of LINC00936 on proliferation, invasion, and migration of EC cells were examined by CCK-8 and transwell assays. Colony formation assay was also performed to evaluate the colony forming ability of EC cells. The effect of overexpression of LINC00936 on the expression of EMT-related proteins in EC cells was examined by western blot. In addition, the effect of LINC00936 overexpression on the growth of EC *in vivo* was examined by using tumorigenicity assay in the nude mouse.

**Results:**

LINC00936 was expressed at a low level in EC tissues and cell lines and significantly correlated with tumor size and lymphatic metastasis of patients. Overexpression of LINC00936 significantly inhibited the proliferation, invasion, and migration, as well as colony formation ability of EC cells. Western blot analysis showed that overexpression of LINC00936 significantly promoted the expression of E-cadherin and inhibited the expression of N-cadherin and vimentin in EC cells. Tumorigenic assays in the nude mouse demonstrated that overexpression of LINC00936 inhibited the growth of EC *in vivo* by suppressing Ki-67 and promoting E-cadherin expression.

**Conclusion:**

LINC00936 was expressed at a low level in EC tissues and significantly correlated with tumor size and lymphatic metastasis of patients. Overexpression of LINC00936 significantly inhibited the proliferation, invasion, and migration, as well as colony formation ability of EC cells. Therefore, LINC00936 could be a new target for the early diagnosis and treatment of EC patients.

## 1. Introduction

Endometrial carcinoma (EC) is one of the three most common malignant tumors in the female reproductive system, accounting for about 20%–30% of female reproductive system malignant tumors and 7% of female systemic malignant tumors [1]. In recent years, its incidence has been increasing, and the age of the onset is becoming younger, which poses a serious threat to women's health [2]. With early surgery and comprehensive treatment of this tumor, patients generally have a good prognosis and a high 5-year survival rate [3]. However, in advanced or metastatic disease, poor prognosis is a major factor leading to patient death due to the spread of the disease and poor differentiation [4, 5]. Therefore, it is vital to actively explore the molecular mechanisms affecting EC progression and to screen novel biomarkers and therapeutic targets, which are essential to improve the survival rate of EC patients.

Long noncoding RNA (lncRNA) is a class of noncoding RNA greater than 200 nucleotides in length. As the most widespread and heterogeneous non-protien-coding RNA in cells, it is involved in cell proliferation, differentiation, and apoptosis through epigenetic modification and posttranscriptional regulation of genes. LncRNA is also involved in the regulation of tumorigenesis and progression of malignant tumors [6–8]. It has been shown that lncRNAs play important roles not only in the pathogenesis of EC but also in the metabolism, invasion, and metastasis of endometrial tumor cells [9]. For example, lncRNA SNHG14 was highly expressed in EC tissues and significantly correlated with a tumor size, pathological grade, and poor prognosis of patients, and it was able to promote malignant progression of EC by regulating miRNA-655-3P expression [10]. The lncRNA ROR was highly expressed in EC tissues, and overexpression of this gene significantly promoted HEC-1A cell proliferation and inhibited cell apoptosis [11]. In addition, lncRNAs are also involved in regulating drug resistance in EC. For example, lncRNA HEIH enhances the tolerance of EC cells to paclitaxel by activating the MAPK signaling pathway [12]. High expression of lncRNA CDKN2B-AS is associated with adverse response to paclitaxel in EC patients and inhibits resistance to paclitaxel in EC patients *via* the miR-125a-5p-Bcl2/MRP4 pathway [13]. Therefore, it is important to investigate the mechanism of lncRNA in the growth process of EC to reveal the occurrence and development of EC and find new therapeutic targets.

LINC00936 is a newly identified class of noncoding RNA with important physiological regulatory functions. However, there are relatively few studies on how LINC00936 exerts its physiological functions. In 2020, Sheng's group analyzed the relevant differential lncRNAs in acute myocardial infarction by using a transcriptome and found that LINC00936 was involved in regulating the development of the disease process [14]. In addition, Li's group found that upregulation of LINC00936 or silenced microRNA-425-3p suppressed immune escape of gastric cancer cells by elevating the expression of ZC3H12A in 2021 [15]. The above findings suggest that LINC00936 may have an important role in the development of myocardial diseases or tumorigenesis. However, so far, the function and molecular mechanism of LINC00936 in tumor tissues, especially in EC tissues, are unclear. Therefore, this project investigates the expression and biological function of LINC00936 in EC tissues. Our results showed that LINC00936 may play a critical role in the initiation and metastasis of EC, indicating that LINC00936 could be a new marker for early diagnosis and treatment of EC.

## 2. Materials and Methods

### 2.1. Collection of Clinical Samples

This study was approved by the hospital ethics committee, and informed consents were signed by the patients or their families. EC tissue samples were collected from patients who underwent surgery from March 2019 to May 2021. Inclusion criteria: (1) no preoperative radiotherapy and endocrine therapy; (2) clear endometrioid adenocarcinoma on postoperative histopathological examination; and (3) complete clinical data. Exclusion criteria: (1) patients with nonprimary EC and other malignant tumors in combination and (2) patients with serious lesions of vital organs such as the heart, liver, and kidney or immune system diseases. A total of 36 cases, aged 29 to 71 years, with a mean age of (48.48 ± 11.63) years, were included. The EC tissues and the paracancerous tissues more than 5 cm away from the edge of the cancer tissues were retained during surgery. Tissues were put into an RNA buffer and then stored in liquid nitrogen for freezing, and the total RNA in the tissues was subsequently extracted.

### 2.2. Cell Culture

EC cell lines (HEC-1B, HEC-1A, KLE, and Ishikawa) and normal endometrial stromal cells (T-HESC) were purchased from the Shanghai Cell Center, Chinese Academy of Sciences. The above cells were cultured in the RPMI 1640 medium, except HEC-1A cells were cultured in McCoY's 5A medium, and Ishikawa cells were cultured in Eagle's medium, with 10% fetal bovine serum, 100 U/ml of penicillin, and 100 mg/ml of streptomycin. All cells were cultured in a cell culture incubator with 5% CO_2_ and 37°C saturated humidity.

### 2.3. Fluorescence Quantitative PCR Experiments

The frozen EC and paracancerous tissues were taken, cut , and added to 1 mL Trizol for total RNA extraction. The quality of the total RNA was measured using the Nanodrop2000 UV spectrophotometer and stored at −80°C for backup. Primer sequences for LINC00936 and GAPDH are referenced in the literature [15]. The reactions were performed on a fluorescent quantitative PCR instrument using GADPH as a reference gene. Relative quantification of individual genes was performed by a double standard curve. The relative expression of each gene was calculated by the 2^−ΔΔCt^ method, where ΔCt = target gene Ct value−internal reference gene Ct value and ΔΔCt = transfected group ΔCt−control group ΔCt. The expression was analyzed in conjunction with the patients' staging and clinical data to determine the correlation of LINC00936 expression with the clinicopathological characteristics of patients.

### 2.4. Cell Transfection Experiments

The overexpression lentiviral vector pcDNA-936 and the blank control vector pcDNA-control were designed by Shanghai Biotech Biotechnology Company according to the LINC00936 gene sequence. EC cells in the exponential growth phase were collected, counted, and inoculated into 6-well plates and incubated in a CO_2_ incubator at 37°C and 5% CO_2_. Before transfection, the cells were washed twice with a serum-free medium and 9 MOI of virus was added. The plates were then incubated for 2 hours at 37°C in an incubator, with gentle shaking every 15 minutes. Then, 2 mL of the medium was added after which the plates were incubated in the incubator for 48 h.

### 2.5. Cell Activity Assay

The effect of overexpression of LINC00936 on the proliferative capacity of EC cells was examined with the CCK-8 activity assay kit. Firstly, the transfected EC cells were digested and inoculated into 96-well plates with 2000 cells per well and then cultured at 37°C with 5% CO_2_ for 24, 48, 72, and 96 h. 10 *μ*L of the CCK-8 solution was added to each well, and the plate was incubated for 4 h. The absorbance 450 nm was then measured.

### 2.6. Colony Formation Assay

A colony formation assay was used to detect the effect of overexpression of LINC00936 on colony-forming capacity of EC cells [16]. Transfected cells in the logarithmic growth phase were taken, digested, and diluted into individual cells with a medium containing 10% fetal bovine serum and inoculated with a 2 mL of the medium (containing 600 cells) per well in a 6-well plate and gently rotated to disperse the cells evenly. The cells were incubated at 37°C in a 5% CO_2_ incubator with fluid changes every 3 d. After 2 weeks, the culture was terminated and the supernatant was discarded and washed 2 times with PBS. The cells were fixed with 4% paraformaldehyde for 15 min, then the fixative was removed, and 2 ml of 0.1% crystal violet staining solution was added for 10 min; then, the staining solution was slowly washed off with running water and air dried. The colonies were photographed and counted for analysis.

### 2.7. Western Blot

Transfected cells at the logarithmic growth stage were washed with a precooled PBS buffer, and 100 *μ*l of precooled RIPA (containing 1 *μ*L PMSF) was added. The cells were lysed on ice for 30 min, and the supernatant was collected by centrifugation and quantified by using the BCA protein quantification kit as described in the literature [17]. Samples were added to the gel wells at 70 *μ*g per lane and electrophoresed at a constant pressure of a 40 V (concentrate)/100 V (separator) until bromophenol reached the edge of the gel. The PVDF membrane was soaked with a 5% skim milk powder at room temperature for 2 h. The primary antibody diluted with 5% BSA was added separately and incubated overnight at 4°C. The PVDF membrane was washed three times with the TBST buffer, and the secondary antibody diluted with 5% BSA was added and incubated at room temperature for 1.5 h. The membranes were developed with the ECL Developer A and B dropwise until the protein bands showed on the strip.

### 2.8. *In Vivo* Experiment

Eight 4–6 week-old male nude mice were purchased from the Shanghai Experimental Animal Research Center and housed in an SPF class animal experiment center, with four nude mice per cage. The animal room was illuminated for 12 h and dark for 12 h daily, alternating light and dark, with relative humidity maintained at (50% ± 10%) and temperature at (22 ± 2)°C, and disinfected regularly with UV irradiation. Male nude mice drank water freely and were given special pellet feed. The cells were washed twice with the PBS buffer and made into a single cell suspension and centrifuged at 1000 rpm for 5 min; the supernatant was discarded, and the cells were resuspended with PBS. The cell counting was performed with a cell counting plate, and the cell density was adjusted to 1 × 10^7^ cells/ml. Four nude mice were selected from each group, and the axillae of the nude mice were disinfected with iodophor. A sterile disposable syringe was used to draw 200 *μ*l of cell suspension, and the syringe was gently shaken to distribute the cells evenly; the cell suspension was injected into the axilla of the nude mice. The nude mice continued to be fed with regular chow and were regularly observed daily for tumor formation of transplanted tumors and weighed for weight and volume. Equation V (volume) = (L × W^2^)/2, where L: tumor length and W: tumor width. The nude mice were executed after 30 d, and tumors were stripped out and weighed. They were then stored at −80°C for extraction of total RNA and protein from the tissues and subsequent analysis.

### 2.9. Statistical Analysis

SPSS 19.0 was used to analyze the data, and the measurement data were expressed as (mean ± standard deviation  ± *s*). Comparisons between groups were performed by the *t*-test or analysis of variance (ANOVA) for normally distributed measures and by the nonparametric rank-sum test for nonnormally distributed measures. *P* < 0.05 was considered a statistically significant difference.

## 3. Results

### 3.1. The Expression of LINC00936 in EC Is Significantly Correlated with the Clinical Characteristics of Patients

**Figure 1:**
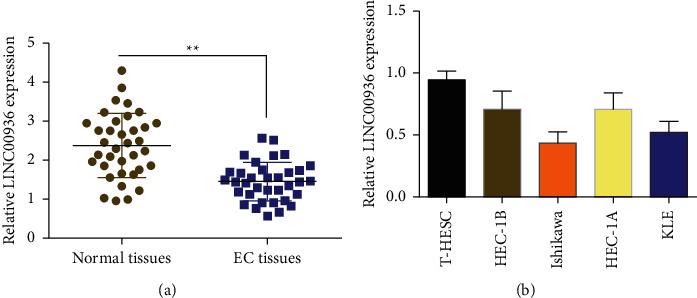
Expression of LINC00936 in EC tissues and cell lines by fluorescent quantitative PCR. (a) Expression levels of LINC00936 in 36 EC tissues and paracancerous tissues by fluorescent quantitative PCR and (b) expression levels of LINC00936 in EC cell lines by fluorescent quantitative PCR, ^*∗∗*^*P* < 0.01.

Endometrial cancer is divided into type I and type II based on its origin, clinicopathological, and genetic features [1]. In this study, the clinical samples used are type I tumors (endometrioid adenocarcinomas). To explore the role of LINC00936 in endometrial carcinogenesis and development, the expression of LINC00936 in 36 endometrial and paraneoplastic tissues was first measured by fluorescence quantitative PCR. The results showed that LINC00936 was expressed at a low level in EC tissues compared with paraneoplastic tissues (Figure 1(a)). Based on the expression level of LINC00936 in EC tissues, we analyzed the relationship between the high- and low-expression of LINC00936 and the clinicopathological characteristics of patients. The results showed that the high expression level of LINC00936 was significantly correlated with tumor size and lymphatic metastasis in EC patients (Table 1). EC cell lines (HEC-1B, HEC-1A, KLE, and Ishikawa) and normal endometrial stromal cells (T-HESC) were examined by fluorescent quantitative PCR, which showed that LINC00936 had lower expression levels in KLE and Ishikawa EC cell lines than in the normal endometrial stromal cells (T-HESC) (Figure 1(b)).

### 3.2. Overexpression of LINC00936 Significantly Inhibited the Proliferation Ability of EC Cells

**Figure 2:**
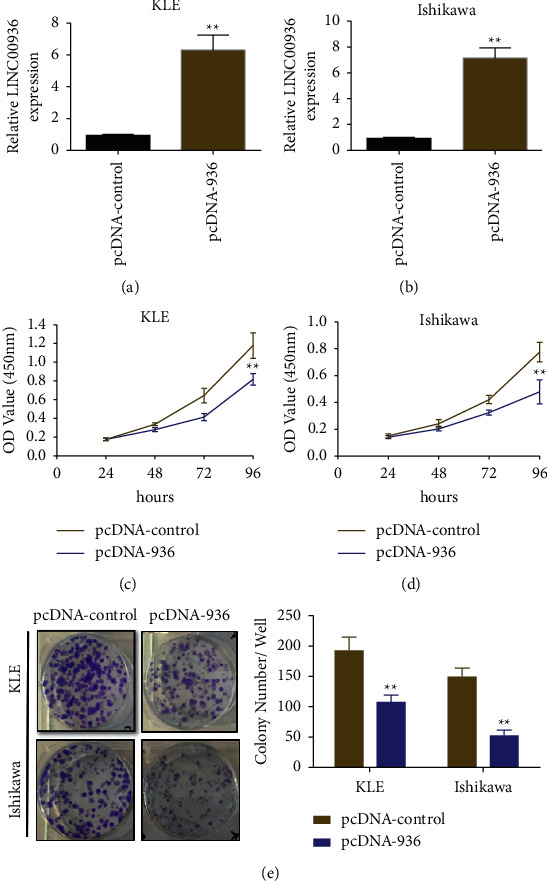
Overexpression of LINC00936 inhibiting the proliferative ability of EC cell lines. (a, b) The expression of LINC00936 detected by fluorescent quantitative PCR in EC cells. (c, d) The effect of overexpression of LINC00936 on the proliferative ability of EC cells detected by CCK-8. (e) The colony formation assay was used to detect the effect of overexpression of LINC00936 on the clonogenic ability of EC cells, ^*∗∗*^*P* < 0.01.

To further examine the specific regulatory function of LINC00936 in EC cells, we constructed an overexpression vector of LINC00936 (pcDNA-936) and a blank control (pcDNA-control) and transfected them into KLE and Ishikawa cells. The fluorescence quantitative PCR assay showed that transfection of the LINC00936 overexpression vector (pcDNA-936) significantly promoted intracellular LINC00936 expression compared with the blank control (pcDNA-control) (Figures 2(a) and 2(b)). The CCK-8 assay showed that transfection with the overexpression vector of LINC00936 (pcDNA-936) significantly inhibited the proliferation ability of EC cells compared with the blank control (pcDNA-control) (Figures 2(c) and 2(d)). The results of the clone formation assay showed that transfection with the overexpression vector of LINC00936 (pcDNA-936) significantly inhibited the colony formation ability of EC cells compared with the blank control (pcDNA-control) (Figure 2(e)).

### 3.3. Overexpression of LINC00936 Significantly Inhibited the Migration and Invasion Ability of EC Cells

**Figure 3:**
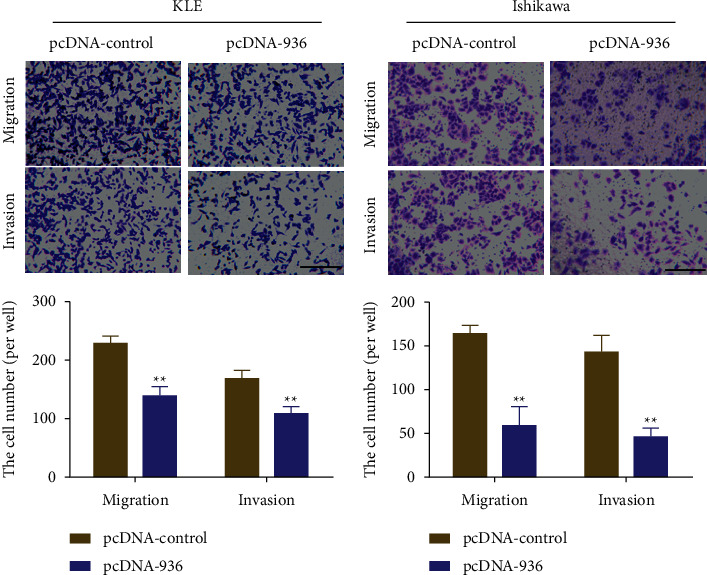
Overexpression of LINC00936 inhibiting the ability of EC cells to invade and migrate, ^*∗∗*^*P* < 0.01, scale bar = 200 *μ*m.

The transwell assay revealed that overexpression of LINC00936 significantly inhibited the migration and invasion ability of EC cells. It was found that transfection of the LINC00936 overexpression vector (pcDNA-936) significantly inhibited the invasive and migratory ability of KLE cells compared with the blank control (pcDNA-control) (Figure 3). In addition, the overexpression vector transfected with LINC00936 (pcDNA-936) also significantly inhibited the invasive and migratory ability of Ishikawa cells compared with the blank control (pcDNA-control) (Figure 3). The above experimental results showed that overexpression of LINC00936 significantly inhibited the migration and invasion ability of EC cells.

### 3.4. Overexpression of LINC00936 Significantly Inhibited the Epithelial-Mesenchymal Transition Ability of EC Cells

**Figure 4:**
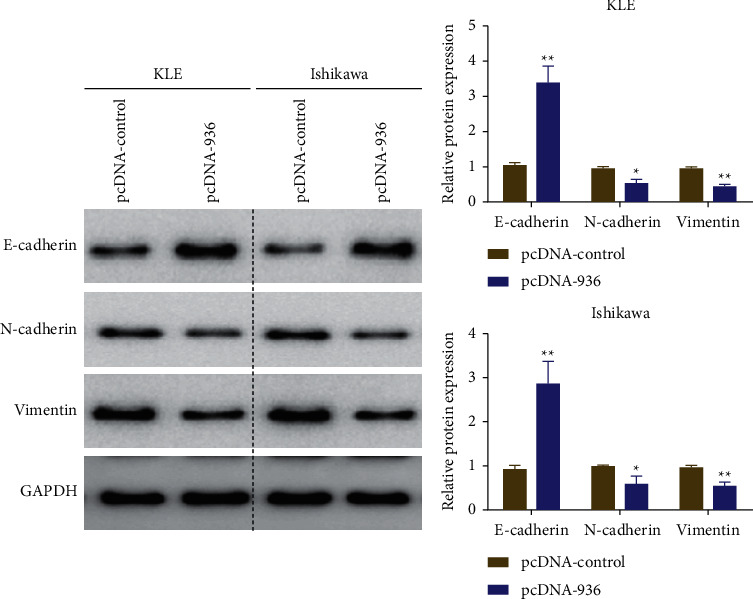
Overexpression of LINC00936 inhibiting the expression of EMT-related proteins (E-cadherin, N-cadherin, and vimentin) in EC cells, ^*∗∗*^*P* < 0.05 and ^*∗∗*^*P* < 0.01.

Activation of epithelial-mesenchymal transition (EMT) has been shown to be a key process in EC cell metastasis, during which epithelial cells acquire mesenchymal cell characteristics and increased cell motility and migration capacity. Therefore, the effect of overexpression of LINC00936 on the expression of key proteins during epithelial-mesenchymal transformation of EC cells was examined by the western blot. The results showed that transfection with the overexpression vector of LINC00936 (pcDNA-936) significantly promoted the expression of E-cadherin and inhibited the expression of N-cadherin and vimentin in KLE and Ishikawa cells compared with the blank control (pcDNA-control) (Figure 4). These results indicate that overexpression of LINC00936 significantly inhibited the epithelial-mesenchymal transition of EC cells and thus inhibited the invasion and migration ability.

### 3.5. Overexpression of LINC00936 Significantly Inhibited the Proliferation Ability of EC Cells *In Vivo*

**Figure 5:**
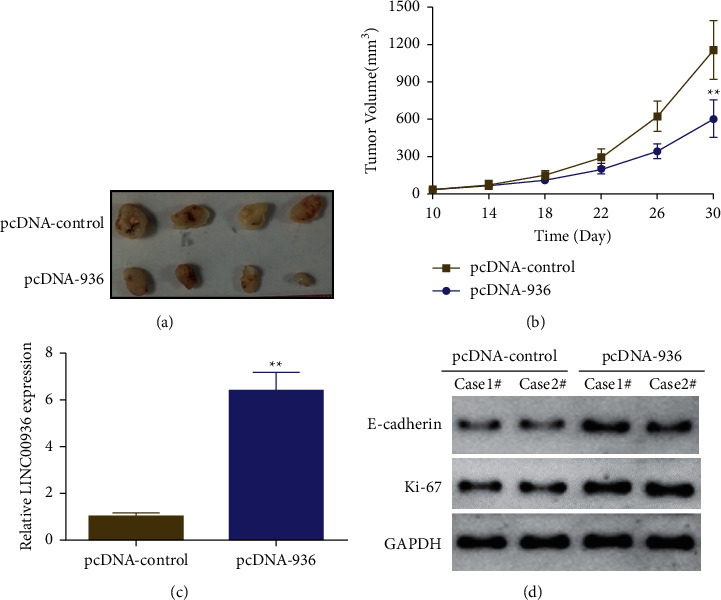
Overexpression of LINC00936 inhibiting the ability of EC cells to proliferate *in vivo*. (a) Representative xenograft tumors for indicated cells. (b) Volume changes of tumors formed *in vivo* after inoculation of EC cells overexpressing LINC00936 in nude mice. (c) The expression of LINC00936 in tumors detected by fluorescence quantitative PCR. (d) Expression of E-cadherin and Ki-67 in tumor tissues by the western blot, ^*∗∗*^*P* < 0.01.

The effect of overexpression of LINC00936 on the growth of EC *in vivo* was investigated using an *in vivo* tumorigenic assay in nude mice. The results showed that overexpression of LINC00936 (pcDNA-936) significantly promoted the EC cell (KLE) tumor growth compared with the blank control (pcDNA-control) *in vivo* (Figure 5(a)). In addition, the results of fluorescence quantitative PCR assay showed that the expression level of LINC00936 was significantly higher in the tumor tissues of the LINC00936 (pcDNA-936) group than that of the blank control group (pcDNA-control) (Figure 5(b)). Western blot results showed that the expression of proliferation-associated protein Ki-67 was significantly lower and that of E-cadherin was significantly higher in the tumor tissues of the LINC00936 (pcDNA-936) group than that of the blank control group (pcDNA-control) (Figure 5(c)). These results indicate that overexpression of LINC00936 significantly inhibited the proliferation ability of EC cells *in vivo*.

## 4. Discussion

EC is one of the most malignant tumors dangerous to women's health, and its incidence is increasing. A total of 81,964 new cases of EC and 16,607 deaths were reported in China in 2020 [18]. Due to the rapid proliferation and migration of EC cells, EC has a high recurrence rate after surgery. Research on the molecular mechanism of EC is important for the molecular-targeting therapy of EC [19]. Current studies have shown that the development of EC is associated with a variety of factors, including genetics, obesity, hypertension, estrogen, and more. [20]. In addition, it has been shown that intestinal flora also regulates the development of endometrial and breast cancer in women by affecting the enterohepatic circulation of estrogen and controlling its secretion [21, 22]. Estrogen might exert its physiological functions through lncRNAs, such as ERINA, an estrogen-responsive lncRNA, which promotes breast cancer development through the E2F1/RB1 pathway [23]. LncRNAs are involved in cell development, proliferation, migration, aging, and other activities, and they have important regulatory functions in the initiation and development of cancers [24]. Therefore, the in-depth study of lncRNA can provide new biological markers for the screening, diagnosis, and prognosis of EC [9]. In this study, LINC00936 was first found to be expressed at a low level in EC tissues and cell lines and significantly correlated with patient clinicopathological features such as tumor size and lymphatic metastasis. Overexpression of LINC00936 significantly inhibited the proliferation, colony formation, invasion, and migration ability of EC cells. The western blot assay showed that overexpression of LINC00936 significantly promoted the expression of E-cadherin and inhibited the expression of N-cadherin and vimentin. The tumorigenic assay in nude mice demonstrated that overexpression of LINC00936 significantly inhibited the growth of EC *in vivo*.

Numerous studies have demonstrated that lncRNAs play a wide range of gene expression regulatory functions in cells, and their abnormal expression can induce disruption of cellular activities and cause tumorigenesis [9, 24]. lncRNA FOXCUT was highly expressed in EC cells. Overexpression of this gene promoted cell proliferation, invasion, and migration as well as epithelial-mesenchymal transition (EMT), inhibited cancer cell apoptosis, and blocked the S-phase cell cycle, whereas silencing of lncRNA FOXCUT had the opposite effect. Therefore, lncRNA FOXCUT has a regulatory role in promoting EC cell progression, providing a new potential target for targeted therapy [25]. In addition, lncRNA can be used as a marker for early detection of EC. For example, lncRNA DLEU1 was significantly elevated in the sera of EC patients and showed good performance in distinguishing EC patients from healthy controls. In addition, the expression of lncRNA DLEU1 in serum was higher in patients with advanced disease than in patients with good clinical features. Patients in the group with high levels of lncRNA DLEU1 expression in serum had worse overall survival than disease-free survival [26]. In this study, LINC00936 was found for the first time to be expressed at a low level in EC tissues and cell lines and significantly correlated with the patient disease stage, suggesting that LINC00936 may serve as a new marker for the early diagnosis of EC.

EC cells are known to proliferate and migrate rapidly, so inhibition of cell proliferation and invasion is the main treatment [3]. LncRNA TDRG1 promotes EC cell proliferation and invasion by regulating the expression of VEGF-A [27], whereas lncRNA ABHD11-AS1 as an oncogene promotes cell proliferation and invasion in EC by regulating the cell cycle protein D1 [28]. The results of the present study are consistent with the above studies, showing that overexpression of LINC00936 significantly inhibited the proliferation and invasive ability of EC cells. In addition, *in vivo* experiments demonstrated that overexpression of LINC00936 significantly inhibited the growth of EC *in vivo*. Although it has been demonstrated that LINC00936 is involved in regulating the development of acute myocardial infarction and immune escape in gastric cancer [14, 15], the molecular mechanism of how LINC00936 inhibits these physiological functions is not clear. Western blot analysis showed that overexpression of LINC00936 significantly promoted the expression of E-cadherin and inhibited the expression of N-cadherin and vimentin. *In vivo* experiments also showed that overexpression of LINC00936 significantly inhibited the expression of Ki-67, a proliferation-associated protein, and promoted the expression of E-cadherin in hormonal tumor tissues, which in turn inhibited proliferation and invasion. However, the molecular regulatory mechanism by which LINC00936 regulates the proliferation and invasion of EC is not clear. In the future, we will continue to explore the upstream regulatory molecules and influencing factors of LINC00936 (such as gut microbial abnormalities or estrogen) and investigate the mechanisms by which LINC00936 regulates downstream molecules and whether it can be used as a marker for liquid biopsy for the early detection of EC.

In conclusion, this study showed that LINC00936 was expressed at a low level in EC tissues and cell lines. *In vitro* cell function assays demonstrated that overexpression of LINC00936 significantly inhibited cell proliferation, colony formation, invasion, and migration. *In vivo* tumorigenic assays in nude mice suggested that overexpression of LINC00936 significantly inhibited EC growth. Our study indicated that LINC00936 has the potential to become a new target for the treatment of EC.

## Figures and Tables

**Table 1:** Association of LINC00936 expression with clinicopathologic characteristics of endometrial carcinoma.

Characteristics	Cases (*n* = 36)	LINC00936 expression		*P* value
		Low	High	
Age				
>60 years	10	6	4	0.463
≤60 years	26	11	15	

Menstruation				
Premenopausal	22	12	10	0.322
Menopausal	14	5	9	

T stage				
T1-T2	21	8	13	0.311
T3-T4	15	9	6	

Tumor size				
≤5 cm	24	8	16	0.033^*∗*^
>5 cm	12	9	3	

Lymph node metastasis				
Negative	20	6	14	0.043^*∗*^
Positive	16	11	5	

^*∗*^Statistically significant.

## Data Availability

The data are available from the corresponding author upon request via email (sunhaizhuhrbmu@163.com).
